# Emergency team competencies: scoping review for the development of a tool to support the briefing and debriefing activities of emergency healthcare providers

**DOI:** 10.1186/s44158-023-00109-3

**Published:** 2023-07-28

**Authors:** Gabriele Lorenzini, Alberto Zamboni, Luca Gelati, Alberto Di Martino, Alberto Pellacani, Nicolò Barbieri, Marcello Baraldi

**Affiliations:** 1 Emergency Department Pavullo N/F, Azienda USL Di Modena, Modena, Italy; 2 Simulation Center SIMANNU, Nuoro, Italy; 3 Azienda USL Di Modena, Modena, Italy

**Keywords:** Debriefing, Briefing, Emergency medicine, Human factor, Non-technical skills, Technical skills

## Abstract

Globally, at least one adverse event occurs in 10% of patients using emergency, inpatient, outpatient, surgical and primary care services. Particularly in emergency health care, this problem is exacerbated by additional variables such as patient criticality, high-risk diagnostic and therapeutic interventions and varying levels of healthcare provider training. In relation to the last point, briefing and debriefing activities during an emergency reinforce staff training and support them in managing work resources, planning interventions and improving future performance. The aim of the scoping review is to explore the state of the art in human factors applied to emergency situations and to develop a new tool to support healthcare professionals in conducting evidence-based briefings and debriefings. This review was developed using a search strategy based on the Arksey and O’Malley’s six-step framework for scoping reviews. The literature analysed and the data identified, which are heterogeneous due to different study methodologies, objectives and types of interventions, suggest that human factors applied to emergency situations are still under-researched. At the end of the data extraction, analysis process, authors’ reviews, discussion rounds and comparison with the multidisciplinary team of healthcare providers, 42 behaviours, 33 elements and 8 domains were considered relevant and included in the *Emergency Team Comptencies* (ETC) briefing and debriefing tool, ranked in order of priority as follows: communication, decision-making, clinical skills, situational awareness, leadership, task management, collaboration and stress and fatigue management. Further research is needed to investigate human factors applied to emergency situations and to generate new evidence to improve clinical practice and reduce the risk of error. In the near future, further studies will be conducted by the authors to test the validity of the Emergency Team Competencies tool in objectively measuring the performance of professionals and multidisciplinary teams.

**Supplementary Information:**

The online version contains supplementary material available at 10.1186/s44158-023-00109-3.

## Introduction

Globally, data from the Organisation for Economic Co-operation and Development (OECD) for the year 2021, show that 10% of patients receiving care in emergency, inpatient, outpatient and elective healthcare services will experience at least one adverse event. There are an estimated 3 million deaths per year due to unsafe care and an extraordinary cost of US $606 billion [1, 2]. In this context, human factors play a crucial role in the development of adverse events, and the results of surveys conducted by the OECD show that only 46% of healthcare professionals feel that they effectively transferred information about patient care during shift changes and during handover between operating units (OUs) [3]. Only 68% of staff report a high level of teamwork within their OU, and that their organization shows an improvement in the quality of care following an adverse event (65%) [3]. This is an indication that patient safety is one of the most important hidden issues for public and private healthcare worldwide [2, 4]. Particularly in emergency healthcare setting, this problem is exacerbated by additional variables such as patient criticality, high emotional involvement of healthcare providers and bystanders, crowded emergency rooms, limited resources, limited time, high-risk diagnostic and therapeutic interventions and different levels of healthcare provider experience and education [5]. As an example, a specific training on the timely use of briefing and debriefing during an emergency may support staff to better manage work resources, planning interventions, recognizing mistakes made, strengths and improving future performance [6].

### Purpose

The research objectives are as follows: (1) to explore the state of the art by means of a scoping review following the six-step framework of Arksey and O'Malley [7] on human factors applied to emergency situations, (2) to create a briefing and debriefing tool that can be applied in clinical reality in emergency situations, consisting of a framework of technical and non-technical observable competencies and (3) to identify the technical and non-technical competencies highlighted in the literature as fundamental in reducing the incidence of adverse events and able to support effective critical patient care. All this competencies should be identified, assessed and discussed with healthcare team members during the briefing and debriefing phases.

### Design

This review was developed using a search strategy based on the Arksey and O’Malley’s six-step framework for scoping reviews [7]. The literature search was conducted in the databases of MEDLINE, PsycInfo, CINAHL, Embase, MedlinePlus, Scopus, Ovid and Google Scholar. The search terms were as follows: (Non-Technical Skills [Title]) AND (Technical Skills [Title]) AND (prehospital) AND (emergency department) OR (Human Factors in emergency) OR (NTS) AND (English[lang]) AND ("2002/01/01" [PDAT]: "2022/10/30"[PDAT]) and TI Non-Technical Skills AND TI Technical Skills AND TI prehospital TI emergency department OR AB Human Factors in emergency AND LA English. The additional search terms for the other databases were human factors in prehospital, human factors in emergency medicine, non-technical skills in prehospital, non-technical skills in emergency medicine, adverse event in emergency medicine and adverse event in prehospital. Studies in the elected literature were manually consulted, and those that met the established criteria were included. Literature from the aviation and aerospace sectors was also analysed, as these are high-risk contexts in which specific and in-depth human factors training and its application are carried out since decades. The inclusion criteria regarded all the original research, primary studies, reports, narrative reviews, systematic review, meta-analyses, scoping reviews, qualitative studies, commentary, textbook and validation studies that delved into, discussed and evaluated at least one domain of technical or non-technical competencies applied to emergency health situations, in the 1995–2022 time frame. All articles written in English were included as well. Literature not comprised in the selected period but considered relevant from a scientific point of view was discussed before being excluded. All studies included were open access, full text or retrievable through university and corporate credentials.

The literature was elected based on the inclusion criteria and the scientific relevance following author comparison sessions. Duplicate literature, lack of access to the full text of the article, non-relevance to the purpose of the study, not published in English or conducted in contexts other than healthcare and aviation/aerospace were not considered.

The literature search was conducted during the period from July 2022 to October 2022. Table 1 is summary of steps related to the literature search.

**Table 1:** Summary of steps related to bibliographic research

Search terms (Non-Technical Skills [Title]) AND (Technical Skills [Title]) AND (prehospital) AND (emergency department) OR (Human Factors in emergency) OR (NTS) AND (English[lang]) AND ("1995/01/01" [PDAT]: "2022/10/30"[PDAT]) TI Non-Technical Skills AND TI Technical Skills AND TI prehospital TI emergency department OR AB Human Factors in emergency AND LA English Human factors in prehospital, human factors in emergency medicine, non-technical skills in prehospital, non-technical skills in emergency medicine, adverse event in emergency medicine and adverse event in prehospital
Databases PubMed, MEDLINE, PsycInfo, CINAHL, Embase, MedlinePlus, Scopus, Ovid MEDLINE and Google Scholar
Inclusion criteria Original research, primary studies, reports, narrative reviews, systematic review, meta-analyses, scoping reviews, commentary, qualitative studies, textbook and validation studies with relevance for non-technical skills and technical skills in the emergency situation, aviation and aerospace sector, publications in English, time frame 1995–2022, open-access publications and studies available through corporate or university credentials
Exclusion criteria Publications not in English, publication not in time frame 2000–2022
Expanded inclusion criteria Literature that was not included in the reporting period and was scientifically relevant was discussed before being excluded
Result 1045 studies, of which 913 were ineligible because they did not meet the inclusion criteria, and the remaining 135 studies were identified as potentially eligible. At the end of the second stage of analysis, 89 studies were excluded because they were either not relevant to the research objective or delved into taxonomies of technical or non-technical skills already included

### Data extraction and analysis

A descriptive-analytical approach, based on qualitative analysis of the technical and non-technical expertise present in the selected literature, was used to maintain a consistent approach to data extraction. The studies were analysed in several stages, starting with title analysis and then with abstract and full-text reading. After the first screening stage, the selected studies were analysed again. The data were entered into extraction tables. (A supplementary film file shows this in more detail. See supplementary file no. 1.) The aim was to summarize and contextualize the main findings and to highlight the technical and non-technical expertise examined. Data extraction was performed by the lead author and reviewed by the other authors using the same process. The following data were extracted: author, publication date, study design, study purpose, setting, human factors investigated and results. Quantitative data found in the selected studies were included to facilitate an understanding of the context and relevance of the technical or non-technical competence examined. The extracted data were discussed in four rounds of virtual meetings at the end of the tabulation review. The aim of the meetings was to identify the relevant competencies for the construction of the briefing and debriefing tool and its application in clinical practice. The internal classification of the tool was divided into domains, elements and behaviours, following the structure of the European Union Aviation Safety Agency (EASA) Competency Framework [8]. Competencies that were eligible at the end of the discussion rounds were discussed again in a fifth round to identify their priority level and position within the framework. (A supplementary film file shows this in more detail. See supplementary file no. 2.) Behaviours and elements included in the framework were discussed in the same rounds, also taking into account techniques, acronyms and practical tools that emerged during the data extraction and review of the results. Disagreements that occurred during the rounds were moderated until consensus was reached in all phases of literature review, data extraction, inclusion and classification of domains, elements and behaviours. The technical and non-technical comptenecies found to be eligible were schematized in a mapping grid (Additional file 4), which included the items found to be eligible for all stages of analysis.

It should be noted that due to the different types of studies and contexts in which the research was conducted, the extrapolated data were heterogeneous.

At the end of the development of the instrument, the optional sixth step described by Arksey and O'Malley [7] was applied, which was to create a network for discussion and comparison with medical health professionals: physicians, nurses and technicians specialized in the management of medical emergencies. The professionals, a total of 13, were physicians specializing in anaesthesia, resuscitation, intensive care and pain management, physicians specializing in emergency medicine, nurses specializing in pre-hospital emergencies, technicians specializing in pre-hospital emergencies and emergency and intensive care nurses. Discussions with clinical experts provided additional views and experiences that encouraged the authors to include further literature and to revise the classification of the briefing and debriefing tool.

## Results and discussion

The first screening identified 1045 studies, of which 913 were ineligible because they did not meet the inclusion criteria, and the remaining 135 were identified as potentially eligible. At the end of the second stage of analysis, 89 studies were excluded because of their lack of relevance to the research objective or because of their repetition of technical or nontechnical knowledge already included. Of the remaining 46, 13 were reviews, 11 were reports, 6 were observational studies, 4 were validation studies, 2 were qualitative studies, 2 were scoping reviews, 2 were textbooks, 2 were systematic reviews, 1 was a mixed methods study, 1 was a cross-sectional study, 1 was a commentary and 1 was a trial. Thirty-three studies were conducted in the health sector, 8 in the aviation sector and 3 in the aerospace sector.

At the end of the data extraction, analysis process, authors’ reviews, discussion rounds and comparison with the multidisciplinary team of healthcare providers, 42 behaviours, 33 elements and 8 domains, ranked in order of priority: *communication*, *decision-making*, *clinical skills*, *situational awareness*, *leadership*, *task management*, *collaboration and stress and fatigue management*, were considered relevant and included in the *Emergency Team Comptencies* (ETC) briefing and debriefing tool.

### Technical skills vs. non-technical skills

Peltonen, Peltonen and Salanterä evaluated the association between non-technical and technical skills in real advanced life support (ALS) scenarios in a hospital setting. The results suggest that they are not independent skills but have a positive association with each other [9]. In this regard, the Emergency Team Competencies tool integrates “clinical skills” within it to overcome the concept that often separates technical and non-technical skills by introducing a single skills framework. All clinical and non-clinical expertise are to be considered “competencies” that every professional must possess and must be appropriately trained.

### Communication between professionals during emergencies

The most common errors recorded by the Safety Human Incident & Error Learning Database (SHIELD) were attributable to staff’s inappropriate approach to other professionals surrounding them [10]. Evans, Evans, Slack, Peddle and Lingard point out that the circular communication is a good strategy for understanding whether the receiver has understood and received the information. Gradual assertiveness was found to be effective when disagreements were present within the work team. The results show that this is important in situations in which the younger professional has to assert his or her point of view against experienced practitioners [11].

From a clinical risk management perspective, the implementation of protocols of assertive communication standardized has reduced the loss of important data, shortened the time to transfer it between professionals and enabled the sharing of mental models [12, 13]. There is no gold standard model in the literature, but the evidence suggests that operating units belonging to the same network adopt a uniform, single method [14, 15]. Standardized communication models, circular communication and feedback are emerging as essential tools for delivering quality care and reducing errors in emergency situations [16]. In this regard, in the civil aviation environment, the regulations reference issued by the European Aviation Safety Agency (EASA) suggests that the communication should be by appropriate means with respect to the operational context, and the information sharing must follow the same procedures to avoid distortion of the message transmitted through telemedicine tools [17].

During the briefing and debriefing of teams, communication should be used to establish the right atmosphere of collaboration, assign roles and responsibilities, settle doubts, requests and defuse emotions or discomfort experienced during operations [18]. Kohn L. T. et al. highlight that effective communication fosters the creation of a universal learning, where professionals are incentivized to let information flow freely, regardless of the degree of authority. All of this constitutes a moment of professional growth that stimulates each member of the team to improve their own performance [19, 20].

### Interprofessional relationships and the role of leadership

Dagnell A. J. pointed out that leadership can improve the critical phases and quality of cardiopulmonary resuscitation, one of the major emergencies faced by healthcare providers [21].

Herzberg, Hansen, Schoonover, Skarica, McNulty, Harrod, Snowden, Lambert and Guise examined the correlation between measured teamwork and observed adverse events in the out-of-hospital setting. Logistic regression analysis showed that the odds of occurrence of adverse events decreased by 28% for each point gained on the Clinical Teamwork Scale (*OR* 0.72, 95% *CI* 0.59–0.88) [22]. A characteristic that determines good teamwork is the social climate, which falls within the responsibilities of all professionals involved, and the key to increase levels of team effectiveness is to optimize that climate [23–26].

The studies underline the importance of staff familiarity with various leadership styles especially when facing a rapidly-changing situation, which requires a corresponding rapid change in leadership style. This is imperative in order to ensure a favourable outcome for the patient, with a subsequent increase in job satisfaction for all practitioners involved [17, 27, 28]. Furthermore, conflicts are universally recognized as one of the causes of delays within healthcare facilities, and they affect significantly on the social climate of the team [29]. At this juncture, the leader represents a key figure in managing these kinds of events [11, 29–32]. The briefing phase, in which the leader may acts as moderator, aims to plan activities, analysing the own skills and the situation in which they are going to operate [10, 11, 33].

### Task management and cooperation

The debriefing phase aims to discuss the services provided, the behaviours used and any errors made, helping to alleviate some conflicts and motivate the team to overcome the fear of negative repercussions [11, 20, 34, 35]. The sharing of goals, information and priorities should be of primary importance in setting a common course of action, encouraging “shared mental models” [36]. In addition, by preparing the team to act according to a predefined pattern, it is possible to identify any issues that can be resolved even before the team is called to action; understaffing situations, the need for multiple resources (human and instrumental) and discussion of any doubts with respect to one’s role/task can be corrected quickly, reducing the risk of error [37, 38].

Relevant in this regard is the management of workloads, with the aim of maintaining a good balance [39] and avoiding the overload of work for someone and inactivity for others. Cummings, Tate, Lee, Wong, Paananen, Micaroni and Chatterjee argue that the implementation of these models of physical and mental preparedness and the spread of a collaborative climate reduce dissatisfaction among staff and incentivize skill enhancement [28] while maintaining high levels of operational flexibility and adaptability [13, 40].

The literature has found positive correlations between effective communication, leadership, cooperation and organization [41, 42]. These non-technical competencies are found to be dependent variables with respect to the others. Insufficient performance of one of them tends to affect the social climate of the team. To maximize cooperation among professionals, studies highlight the need to provide support whenever it is necessary, including providing feedback with respect to the service provided and behaviours adopted. It is also important to be able to accept or request such support when needed, using effective communication models that discourage judgement [12, 23, 42].

### Situational, environment and time awareness

Improved situational awareness and cognitive flexibility result in less stress, less fatigue and a lower incidence of unsafe behaviour by care teams [43]. They also play a role in the previously mentioned areas, particularly communication, shared mental models and confrontational methodologies focused on problem-solving [42]. The English Civil Aviation Authority (CAA) classifies the situational awareness into three levels of awareness: information gathering, understanding and analysis and anticipation [44]. Applied to the healthcare environment, situational awareness results critical in determining whether any intervention is necessary and anticipating future developments of the situation [17, 23, 31].

The World Health Organization includes two other aspects to consider: the self-awareness, based on the analysis of one’s skills, knowledge and limitations imposed by the training acquired and awareness of the team with which one works, which includes the analysis of the skills of each member. Such knowledge promotes the proper allocation of human resources, managing staff appropriately according to the difficulties that may be encountered [38, 45].

Scene assessment (environmental safety), including inherent distractors and hazards, should be done systematically to avoid superficial analysis of the environment [39, 46, 47]. The well-known phenomenon of frequent misalignment is between “chronological time” and “perceived time” by an individual, which is often conducive to the “fixation on the task” behaviour at the expense of maintaining awareness of the big picture of ongoing activities. Bennett R. et al. emphasize that good awareness related to time management helps reduce potential errors in fixation [39].

### Decision-making and clinical skills

The decision-making area aims to promote a standardized patient approach and a thinking methodology that focuses on the rational interpretation of information. Fletcher, Flin, McGeorge, Glavin, Maran and Patey included the cognitive active flow (CAF) model by simplifying decision-making at critical moments, including the steps as follows: situation assessment, problem identification, diagnosis formulation and risk assessment [37, 38]. In the field of civil aviation, the reference regulations issued by the European Union Aviation Safety Agency (EASA) recommend the use of decision aids to reduce the risk of error and the occurrence of cognitive bias [17]. Sedlár M. and Kaššaiová Z. included three additional steps: implementation of the decision, reassessment of it and maintenance of standards [30]. With reference to the standards, clinical practice guidelines (CPGs) aim to improve the process of decision-making by healthcare professionals, clearly describing the scientific evidence and the most effective diagnostic-therapeutic pathways to be undertaken [48].

Although the intuition of the experienced practitioner is not to be systematically excluded, especially when he/she has gained sufficient experience to recognize signs/symptoms related to an immediate risk to the patient’s survival, the systematic approach and adherence to guidelines should be applied by all staff, especially those with limited experience, in order to facilitate the delivery of safe care, evidence-based care and the building of sound training for future practice [30].

This is also supported by the World Health Organization, which recommends the use of models for decision support, data mining and predictive models to support clinical reasoning [38]. The DECIDE model (D: define the problem, E: establish the criteria, C: consider all the alternatives, I: identify the best alternative, D: develop and implement a plan of action, E: evaluate and monitor the results) provides a resource for healthcare executives to make more performance [49]. T-DODAR (T: time, D: diagnose O: options, D: decision, A: act the decision, R: review periodically) is a decision-making tool used in the aviation industry and later applied to the surgical setting [50]. CIRCLE (C: clinical analysis; I: identification and treatment; R: resolution — Ri evaluation; C: consequences and developments management; L: location and transport; E: evaluation in continuous), a decision support model developed by the authors, is a decision-making model under development that could support the reasoning clinical professionals in emergency situations.

Sterling M. R., in the space flight resource management (SFRM) model, defines decision-making as a critical element because its fundamental steps create a more situation more effectively. Each step, therefore, is referred to as a “window of awareness” [45, 51]. Hall, Robertson, Rolfe, Pascoe, Passey and Pit tested the effectiveness of Emergency Protocols Handbook, a medical emergency management and treatment protocol, in a simulated emergency room (ER) environment simulated, observing team error rates. The authors highlight that by applying the manual, physicians are more than halved their teams’ error rates. All groups in the study reduced error rates by at least 20% when they had access to the Manual of Emergency Protocols. Overall, there was a 54% reduction in errors in clinical practice carried out in high fidelity simulation [52].

### Workload, stress and fatigue management

The ability to better manage non-technical aspects in an emergency is an important part of the preparedness of providers to deal with increasingly challenging events of longer duration and frequency [43]. The management of fatigue and stress is closely associated with situational awareness, communication and the social climate that are established in a team. Flowerdew, Gaunt, Spedding, Bhargava, Brown, Vincent and Woloshynowych identified the main factors of stress for emergency department (ED) personnel by analysing the positive and negatives associated with working under pressure and investigating interventions that could improve the team functioning. It was found that leadership, teamwork and emotional intelligence were found to be mediating factors between objective stress and subjective stress, reducing the mental impact on professionals and students [53, 54].

## Conclusion

The literature analysed and the data identified, which are heterogeneous due to different study methodologies, objectives and types of interventions, suggest that human factors applied to emergency situations must deserve great attention and are still under-researched. The proposed briefing and debriefing tool identifies a framework of core competencies to be observed, discussed and assessed with the team and consisting of 8 domains, 33 elements and 42 behaviours. Further research is desirable to deep the investigation on human factors involved into emergency situations and to generate new evidence to improve clinical practice and reduce the risk of error. In the near future, the authors will conduct further studies in order to test the validity of the Emergency Team Competencies tool in objectively measuring the performance of professionals and multidisciplinary teams.

### Implications for practice

The Emergency Team Competencies tool (Table 2) needs periodic revision to update the items as the scientific literature evolves. Also, the experience of personnel who work in emergency situations on a daily basis is considered a relevant contribution to the revision process. In this sense, the tool has already been proposed in various trainings with advanced simulation for healthcare personnel prior to its current drafting, with the intent of identifying possible errors and improving comprehensibility. These training experiences convinced the authors that for effective implementation of the Emergency Team Competencies tool within departments, it is necessary to conduct in advance some training sessions dedicated to the methodologies in applying the tool in clinical practice. A working version of the Emergency Team Competencies tool is included in supplementary file no. 3 to facilitate its use in daily clinical practice.

**Table 2:** Emergency team competency tools (ETC tools)

Areas	Elements	Behaviours
Communication	**Listening** **Clarity and relevance “Close loop”** **Feedback loop** **Standard phraseology** **Assertiveness**	• Demonstrates willingness to listen with empathy • Makes sure that the recipient is ready and able to receive information • Delivers messages clearly, comprehensibly, concisely, comprehensively, correctly • Confirmation is expected after making a request or giving orders • It requests and provides the return messages after each communication • Uses acronyms, abbreviations, sequences, etc • Maintains his point of view respectfully (doubt, worry, request, action) • Activates to limit non-essential talks and interruptions in critical situations
Decision-making	**Diagnosis** **Options** **Risk assessment** **Review**	• Involves the team periodically in the diagnosis process • Decides about the action plan (stay and play; scoop and run) • Evaluates different options (risks and benefits) and considers alternative plans • Periodically reviews the decision made and is available for changes • Uses decision aids
Clinical skills	**System awareness Compliance with standards Knowledge and application of procedures/guidelines Understanding of legal regulations** **Quick look**	• Shows familiarity with systems, their limits and interactions • Able to locate each item of the equipment within the operational space • Knows and applies procedures, guidelines, algorithms and international standards • Justifies to colleagues any deviation from established standards • Demonstrates adherence with the rules of conduct, applicable regulations and legal responsibilities • Recognizes obvious clinical signs; interprets posture, non-verbal and para-verbal language to establish the patient’ status
Situation awareness	**Information gathering** **Understanding and analysis** **Anticipation**	• Actively searches for information related to the following: patient status, environment, timing • Organizes information and analyses it critically • Verifies the assumptions made and is willing to update the “mental model” • Identifies distracting factors (interruptions, noise, etc.) • Anticipates possible developments and consequences of actions
Leadership	**Leadership style** **Briefing and planning** **Conflict management**	• Adapts leadership to the situation and the group • Shares the vision, goals, thoughts and actions with others to achieve better results • Performs briefing and debriefing • Involves colleagues in planning and coordinating activities • Calm in conflicts and able to negotiate alternative solutions
Task management	**Priorities setting** **Time optimization** **Resource management** **Task distribution** **Role adherence**	• Sets the right priorities and is flexible in changing them • Considers time constraints/deadlines • Identifies and uses all available resources (human and otherwise) • Balances the workload within the team • Knows the assigned tasks and the associated limitations
cooperation	**Coordination and integration** **Supporting others** **Sharing** **Call for help**	• Collaborates actively with colleagues; shows adaptability • Is attentive to colleagues’ work, helps if necessary • Asks for help promptly when needed • Shares information, doubts and concerns • Knows how to give and receive feedback honestly and respectfully
Stress & fatigue management	**Stress factors** **Emotions** **Coping strategies**	• Recognizes sources of stress and the effects on oneself and on team members • Shares own psychophysical condition with the group (emotional debriefing) • Has good management of personal stress and emotions

## Supplementary Information

**Additional file 1.** Data mining of the literature included at the end of the first screening.

**Additional file 2.** Mapping of behavioural markers broken down in the ETC tool.

**Additional file 3.** Emergency Team Competencies tool.

**Additional file 4.** Mapping of the behavioural markers, elements and competence domains explored in the included studies and multistage development of the ETC tool.

## Data Availability

The data collected are available in the manuscript itself. The additional material was included in the supplementary files published with the manuscript.
