# Supplementary material for: Emergency team competencies: scoping review for the development of a tool to support the briefing and debriefing activities of emergency healthcare providers
Source: J Anesth Analg Crit Care. 2023 Jul 28;3:24. doi: 10.1186/s44158-023-00109-3 (PMC10386683; doi:10.1186/s44158-023-00109-3)
Supplement: Supplementary file 1 — Additional file 1. Data mining of the literature included at the end of the first screening. [file 44158_2023_109_MOESM1_ESM.docx]

| **N°** | **Authors** | **Publication date** | **Study design** | **Study purpose** | **Setting** | **Human factors investigated** | **Results** |
| --- | --- | --- | --- | --- | --- | --- | --- |
| 9 | Peltonen, V., *et al.* | 2020 | Pilot study | Evaluate the association between non-technical and technical skills in real Advance Life Support situations in Emergency Room. | Emergency Department | Non-Technical Skills (NTS) and Technical Skills (TS) | This study presents an association between NTS and TS for the first time in real-life in-hospital Advance Life Support situations. Our results suggest that NTS and TS are not independent skill sets but have a positive association with one another. |
| 10 | DBL. UoS. ECTL. NLR. MAI, RSSB. ENAC. NTUA | 2020 | Report | Identification of requirements for the definition of the Safety Human Incident & Error Learning Database Taxonomy (SHIELD). | Aviation | Management, planning, communication, briefing | Identify non-technical areas for disaster analysis, coordination, planning, communication and instructions, control room communications, material control, radar monitoring, briefing and handover, acquisition, training, supervision, control. |
| 11 | Evans, J.C., *et al.* | 2021 | Scoping review | Provide future researchers and educators with a clearer understanding of team dynamics and a common language for NTS, particularly in pre-hospital resuscitation, first aid and trauma teams. | Emergency Department | Debriefing, mental readlines, adabtive behaviours, share mental model. | Areas of leadership, communication, teamwork, briefing/planning, resource management, stress/fatigue management, followership, debriefing, decision making, situational awareness, team situational awareness, mental readlines, adaptive behaviours, shared mental model were identified.  Proposed taxonomy of non-technical skills for resuscitation teams, including  - Leadership  - Communication skills  - Teamwork  - Planning and briefing  - Resource management  - Stress and fatigue management  - Followership  - Debriefing  - Decision-making  - Situational Awareness / Team Situational Awareness  - Mental preparation  - Adaptive behaviour  - Shared mental models |
| 12 | Steinemann, S., *et al.* | 2012 | Validation study | Evaluate the reliability and correlation with clinical performance of the T-NOTECHS instrument. | Emergency Department | Leadership, cooperation and resource management, communication, evaluation and decision-making, situational awareness/stress management | The real-time inter-rater reliability for assessors with different backgrounds was moderate, but the inter-rater reliability for the teamwork experts who performed the video review was better. The significant improvement in T-NOTECHS scores after teamwork training and the correlation with clinical parameters in simulated and actual trauma resuscitations suggest the clinical relevance of the tool.  Five non-technical skill areas were investigated to improve team management performance:  - Leadership  - Collaboration and resource management  - Communication  - Assessment and decision making  - Situational awareness / stress management |
| 13 | Hamilton, A.L., *et al.* | 2018 | Mix method | Develop a system of behavioural markers specifically applicable to medical students in simulation scenarios. | Acute care | Communication, teamwork, situational awareness, decision making, task management. | The Medi-StuNTS tool was found to be useful in assessing the performance attributable to non-technical skills in medical students.  The literature review phase identified 8 areas of non-technical skills applicable to the emergency setting:  - Communication  - Teamwork  - situational awareness  - Decision making  - Coping with stress  - Role awareness/boundary awareness  - Task management  - Miscellaneous |
| 14 | Donaldson, L. *et al.* | 2021 | Textbook | Examine the state of the art in patient safety and provide theoretical and practical models for clinical risk management. | Interdisciplinary | Decision making, situational awareness. | Facilitate the decision making process by incorporating the steps of situation assessment, problem identification, diagnosis formulation, risk assessment.  Increased situational awareness through the implementation of communication models aimed at organising the information gathered. |
| 15 | Golling, M., *et al.* | 2022 | Validation study | To develop a validated tool to measure the quality of inter-professional communication and relationships during handover in the out-of-hospital context. | Prehospital | Communication, teamwork. | The ED-HFH has demonstrated feasibility, reliability and validity as a measure of the quality of information transfer and human factors in handovers between ambulance services and emergency departments. |
| 16 | Kim, Y., *et al.* | 2020 | Systematic review | Systematically examine whether the clinical outcomes reported in the literature support telemedicine decision support for medical interventions in pre-hospital care. | Prehospital | Communication. | Telemedicine's ability to share information provides access to remote experts to support medical decision making at the point of care or in extended care in the field. |
| 17 | EASA | 2021 | Report | EBT Skills Development and Recommendations | Aviation | Communication, leadership and teamwork, problem solving and decision making, situational awareness, information management, workload management. | Ideal behaviour for dealing with emergency situations and flight management:  - Focus on communication of information within the team  - Leadership models and styles applicable to civil aviation  - Implementation of decision support tools (T-DODAR), standardisation of decision making and adaptation  - Situational awareness and behavioural markers  - Workload management |
| 18 | Purdy, E., *et al.* | 2020 | Commentary | To apply a macro-organisational theory, relational coordination, to a micro-issue, the care of a single patient, to explore team briefing. | Emergency Department | Briefing | Deliberate efforts to target briefings to maximise the rapid development of relational coordination at the micro level should be a priority. This critical step can be incorporated into the ZPS and reiterated throughout resuscitation, as appropriate, to optimise non-clinical processes and facilitate ongoing coordination of care. |
| 19 | Kohn, L. T., *et al.* | 2000 | Textbook and report | Deepen the topic of safety in healthcare. | Interdisciplinary | Communication, leadership | Direct and indirect models and modes of intervention have been extrapolated to improve the safety of the services provided.  Respect human limits:  - Work safely.  - Avoid relying on memory.  - Use constraints and forcing functions.  - Avoid relying on alertness.  - Simplify key processes.  - Standardise work processes.  Encourage effective teamwork:  - Train those who need to work in teams.  - Involve the patient in the safety design and care process.  Anticipate:  - Take a proactive approach: assess care processes for safety threats and redesign them before incidents occur.  - Improve access to accurate and timely information.  Create a learning environment:  - Encourage reporting of errors and unsafe conditions.  - Ensure that there is no retaliation for reporting errors.  - Develop a work culture where communication flows freely, regardless of level of authority.  - Establish mechanisms for feedback and learning from mistakes. |
| 20 | Landon, L. B., *et al.* | 2018 | Report | Discuss the challenges of effective teamwork in a Mars mission scenario. | Space Flight | Communication, conflict resolution. | HERA crews and astronauts aboard the ISS report that appropriate affiliative humour is a key factor in crew compatibility, conflict resolution and overcoming difficulties.  Establishing clear and overarching goals, building trust between teams prior to launch, establishing and training communication norms, and debriefing can help mitigate some conflicts and motivate teams to overcome fear of negative repercussions. |
| 21 | Dagnell, A.J. | 2020 | Report | Reinforce the theme of teamwork and leadership. | Prehospital | Leadership, teamwork. | The non-technical skills of teamwork and leadership can improve the quality of cardiopulmonary resuscitation, which is associated with improved survival outcomes. |
| 22 | Herzberg, S., *et al.* | 2019 | Observation study | To examine the relationship between measured teamwork and adverse events in the pre-hospital care of children using high-fidelity simulation. | Prehospital | Teamwork. | Logistic regression analysis adjusting for clustering at the team level showed that the odds of an error decreased by 28% with each unit increase in CTS (OR 0.72, 95% CI 0.59 to 0.88). This study found that overall teamwork among care teams was strongly associated with the risk of serious adverse events in simulated scenarios of care for critically ill and injured children. |
| 23 | Civil Aviation Authority | 2014 | Review | Provide practical and structured guidance on CRM training for commercial airlines. | Aviation | Situational awareness, workload management | Identification of 3 levels of situational awareness  - Perception of the basic elements  - Understanding their significance  - Projection of their impact in the near future  Prioritisation in terms of workload management:  - Reduction of concurrent or serial tasks  - Switching continuously from one task to another  Identification of NOTECHS applicable to civil aviation:  - Collaboration  - Leadership and management skills  - Situational awareness  - Decision making process  Identification of 14 elements from the previously described areas, such as team building and maintenance, consideration of others, supporting others, conflict resolution, use of authority and assertiveness, maintaining standards, planning and coordination, workload management, systems awareness, environmental awareness, anticipation, problem definition, generating options, risk assessment and option selection. |
| 24 | Boamah, S.A. *et al.* | 2017 | Predictive cross-sectional survey | To investigate the effects of nurse managers' transformational leadership behaviours on job satisfaction and patient safety outcomes. | Management | Trasformational leadership | The transformational leadership component, inspirational motivation, had the strongest impact on nurse and patient outcomes, while individualised consideration was the lowest ranked factor. Through inspirational motivation, transformational leaders communicate high expectations to followers, which inspire them to engage and participate in efforts to achieve the shared vision in the organisation.  A leader who practices transformational leadership emphasises the benefits of collaboration, creating a culture where dialogue is open and new ways of thinking are encouraged. Such leaders empower nurses to solve problems, influence changes in practice on their units, and take ownership of patient care, which can lead to fewer errors. |
| 25 | Sharon, J. | 2018 | Narrative review | To compares transformational leadership and ethical leadership. | Interdisciplinary | Transformational leadership | Ethical and transformational leadership styles have their weaknesses and strengths, but overall they are both seen in a positive light.Transformational leadership style can improve teamwork, patient care, patient satisfaction, staff satisfaction, employee functioning and retention of new nurses. Ethical leadership can improve employee behaviour, relationships and employee and organisational performance.  Transformational leaders who are morally and ethically strong inspire a positive culture and achieve positive results. For transformational leaders to bring about much needed change in people and organisations, the process should include moral and ethical behaviour such as being honest and treating others with dignity and respect. |
| 26 | Lo, D., *et al.* | 2018 | Narrative review | To provide an overview of the key concepts and weaknesses of transformational leadership theory and discuss its relevance in the context of the NHS working environment. | Interdisciplinary | Transformational Leadership | Transformational leadership, like earlier 'great man' theories, places too much emphasis on the importance of the 'heroic' leader. It assumes that influence is unidirectional, flowing from the leader to the followers. Thus, depending on whether the leader is ethical or not, followers who succumb to the allure of a transformational leader risk realising the leader's vision, however impractical, overambitious or deceptive it may be.  Transformational leadership may not be effective in situations where followers do not yet have the skills or competence to complete tasks independently. |
| 29 | Saltman, D.C., *et al.* | 2006 | Narrative review | Identify the difference between conflict and disagreement and describe the nature of conflict in the health care system. | Interdisciplinary | Conflict management | While conflict cannot always be avoided, much can be done to minimise its destructive effects and maximise its constructive outcomes. Preparation for post-conflict interaction is essential. Use goal-setting techniques to ensure that your goals are short-term and flexible enough to keep a dialogue open with the other party.  Remember the background to the conflict: how it happened and why. Often these factors predate the actual event. You should be aware of the 'worst and best case scenarios' and what you are prepared to accept. Rehearsing the negotiation process with a friend or colleague can help to identify options.  Timing, setting and who is present are also important. While it is important to have a safe and neutral environment in which to negotiate a solution, removing the discussion from the setting in which it took place, or having a third party conduct the negotiation, can also have pitfalls. |
| 30 | Sedlár, M., & Kaššaiová, Z. | 2022 | Qualitative study | Identification of cognitive ability markers for prehospital emergency team leaders. | Prehospital | Situational awareness, decision making. | Three elements and 30 behavioural markers have been identified in relation to situational awareness:  - Gathering information  - Interpretation of information  - Anticipating states  3 elements and 20 behavioural markers were identified in relation to decision making:  - Implementing the decision  - Re-evaluating the decision  - Maintaining standards |
| 31 | Flin, R., *et al.* | 2003 | Review | Identification and development of a methodology to assess the non-technical skills of pilots | Aviation | Crisis Resource Management | The areas have been identified:  - Collaboration  - Leadership and management skills  - Situational awareness  - Decision-making process  15 elements were identified:  - Team building and retention  - Considering others  - Supporting others  - Managing conflict  - Using authority and assertiveness  - Setting and maintaining standards  - Planning and coordination  - Managing workload  - Awareness of aircraft systems  - Awareness of external environment  - Awareness of weather  - Problem Definition  - Option generation  - Risk assessment and option selection  - Review of results |
| 32 | Hörmann, H., *et al.* | 1998 | Review | Review and analysis of cultural issues and their impact on crews. | Aviation | Collaboration, leadership and management skills, situational awareness, decision making | On the basis of the empirical results reported, it can be concluded that it is possible to construct assessment methods applicable to European aviation personnel to assess non-technical competencies. The authors suggest that such an assessment can be carried out without significant national bias. The authors point out that the prerequisite is that the methodology is formulated in simple, international English and that the experts are able to communicate in the same English.  Four core areas of non-technical competence have been identified:  - Collaboration  - Leadership and management skills  - situational awareness  - Decision-making  14 elements were identified that belong to the 4 non-technical competence domains:  - Building and maintaining teams  - Consideration and support for others  - Conflict resolution  - Using authority and assertiveness  - Setting and maintaining standards  - Planning and coordination  - Workload management  - System awareness  - Environmental awareness  - Anticipation  - Problem definition/diagnosis  - Option generation  - Risk assessment/option selection  - Review of results |
| 33 | Bleetman, A., *et al.* | 2012 | Review | Discuss solutions to prevent errors and the need for human factors training in emergency medicine. | Emergency Department | Human error, briefing. | The unique operational characteristics of A&E that predispose to human error:  - High patient turnover  - Variety of clinical conditions  - Time constraints  - Heavy reliance on other services, such as radiology and laboratory  - Multiple distractions and interruptions  - Limited history and diagnostic information  - Shift work, different shift times for different groups of staff  - Impaired teamwork  - Rapidly changing and evolving situations  - Involvement of clinicians from other specialties with limited understanding of A&E work  People working in complex systems are more likely to make cognitive errors.  The four triggers for these errors are  - Interruptions and distractions  - Required tasks outside the normal routine  - Unforeseen new tasks  - Interweaving of multiple tasks  Bringing order and structure to an overcrowded department through planning (briefing):  - The current situation  - Who is on the team and their level of experience  - Who is best placed to deal with which patients and crises, so that the most effective use is made of team members rather than a haphazard arrangement.  - Identifying potential pitfalls and threats in advance, such as staff shortages.  - Sharing opinions and concerns. |
| 34 | Andlauer, E., *et al.* | 2001 | Validation study | Operational validation of NOTECHS. | Aviation | NOTECHS | NOTECHS has been successfully validated in the field, albeit on a small scale. Much attention should be given to training in the use of NOTECHS, particularly the calibration and standardisation of trainers.  NOTECHS can provide a 'common culture' to support debriefing and training in non-technical skills. |
| 35 | O'Connor, P. *et al.* | 2002 | Observational study | Development of a European system of behavioural markers for CRM assessment. | Aviation | NOTECHS | The very high level of acceptance and endorsement shown by the instructor groups in the different areas of Europe gives cause for optimism in convincing the aviation community that a method such as NOTECHS could play a valuable role in the quest for higher levels of aviation safety. |
| 36 | Myers, J. A. *et al.* | 2016 | Observational study | Adapt and evaluate a non-technical competency assessment framework for the air ambulance clinical environment. | Prehospital | Task management, teamwork, situational awareness, decision making. | Four domains of non-technical skills adaptable from the ANTS taxonomy were identified (task management, teamwork, situational awareness, decision making).  15 elements belonging to the 4 domains studied were identified with reference to gold standard behaviours that can be implemented by professionals working in the air ambulance clinical environment (planning and preparing, prioritising, maintaining standards, identifying and using resources, coordinating activities with the team, sharing information, using authority and assertiveness, assessing skills, supporting others, gathering information, recognising and understanding, anticipating, identifying options, balancing risks and choosing options, reassessing). |
| 37 | Fletcher, G. *et al.* | 2003 | Review | To present the context in which the Taxonomy and Anaesthetist Behaviour Assessment Tool (ANTS) was developed. | Anaesthesia and resuscitation | Task management, teamwork, situational awareness, decision making. | Identify 4 areas of non-technical skills:  - Task management  - Teamwork  - situational awareness  - Decision making  Identification of 15 elements belonging to the non-technical competence areas  - Plan and prepare  - Setting the right priorities  - Propose and maintain standards  - Identifying and using resources  - Coordinate activities with team members  - Share information  - Use authority and assertiveness  - Assessing skills  - Supporting others  - Gathering information  - Recognising and understanding  - Anticipating  - Identifying options  - Weighing risks and selecting options  - Reassessing |
| 38 | World Health Organization | 2016 | Textbook | Provide information on key issues that may affect safety in the provision of primary health care. | Primary care. | Teamwork, data management and completeness of patient records, patient engagement, decision support | Behavioural markers and activities indicated to increase patient safety levels were extrapolated:  - Task analysis  - Workflow assessment  - Standardised patient care steps  - Team situational awareness  - Prospective risk assessment  - Functional allocation of tasks between team members and technologies  - Understanding the "work" of the patient  - Integration of patients and families into primary care teams, including team training  - Data mining and predictive modelling  - Decision making and decision support  - Prioritisation of diagnostic and therapeutic recommendations |
| 39 | Bennett, R. *et al.* | 2021 | Scoping review | Create the first empirically identified list of non-technical competencies for paramedical and healthcare personnel. | Prehospital | NTS | This review identified the first empirical list of desirable NTS for a paramedic.  26 non-technical paramedic competencies were identified based on empirical data: Decision making, Teamwork, Respect, Adaptability, Communication skills, Situational awareness, Reflection, Non-discrimination, Empathy, Professionalism, Relationship, Mentoring, Leadership Skills, Compassion, Assertiveness, Task Management, Ethical, Interpersonal, Problem Solving, Emotional Intelligence, Dealing with stress, Listening skills, Time Management, Resilience, Scene Management, Integrity. |
| 40 | Moll-Khosrawi, P., *et al.* | 2019 | Validation study | To develop and validate the Anaesthesia Students' Non-Technical Skills (AS-NTS) as a tool to assess students' NTS in emergency and anaesthesia education. | Anaesthesia and resuscitation | Situational awareness, prioritising, decision making, maintaining standards, coordinating team members and activities, communication, leadership, teamwork. | Twelve observable and assessable non-technical skills were identified in anaesthesia students: Situational awareness, prioritising, decision making, maintaining standards, coordinating team members and activities, communication, leadership, teamwork, using authority and assertiveness, team building, resolving conflict/problems, supporting others.  In the focus group sessions with the experts, the following non-technical skills were rated as "very important  o Prioritising (planning task)  o Decision making  o Coordinating team members and activities  o Communication  o Leadership  o Team orientation/teamwork |
| 41 | EASA | 2014 | Report | Regulation of pilot training in the NOTECHS framework for aviation and in-flight activities. | Aviation | NTS | Common situational awareness; common information gathering and processing; workload management; effective communication and coordination between all crew members, including flight crew and inexperienced cabin crew; leadership, cooperation, synergy, delegation, decision making, actions; developing resilience; surprise and startle effect; cultural differences; identifying and managing passenger human factors: crowd control, passenger stress, conflict management, medical factors. |
| 42 | Hayes, P., *et al.* | 2021 | Narrative review | Develop a taxonomy of non-technical skills useful for emergency response teams. | Prehospital | Communicating, Coordinating, Collaborating, Leading, Awareness, Decision-making | Differences at elementary and behavioural levels highlight the need to adapt existing NTS models for emergency services. The ability to better manage non-technical aspects of emergencies is an important part of operators' preparedness to deal with increasingly challenging, prolonged and frequent events.  Seven non-technical competence areas have been identified:  - Communication  - Coordination  - Collaboration  - Leadership  - situational awareness  - Decision-making skills  - Coping, stress & fatigue management  16 elements were identified that belong to the non-technical competence areas:  - Effective communication  - Proactive communication  - Clarifying roles, responsibilities and expectations  - Adapting to demands  - Contributing to a positive team environment  - Aligning efforts and managing conflict  - Creates an appropriate team environment  - Provides focus, direction and coordination  - Gathers and analyses information  - Identifies contingencies, issues and expectations  - Share information and insights  - Informed, timely decisions  - Appropriate approach to decision making  - Involving others in decision making  - Manages pressure  - Uses effective coping strategies - Uses effective coping strategies |
| 43 | Sedlár M. | 2022 | Observational study | Investigate the relationships between work-related factors - stress and fatigue, cognitive skills - situational awareness and cognitive flexibility, unsafe behaviour and involvement in safety incidents among emergency physicians. | Prehospital | Situational awareness, cognitive flexibility. | Improved situational awareness and cognitive flexibility are associated with reduced stress, fatigue, frequency of unsafe behaviour and involvement in safety-related incidents among EMS crew members. |
| 45 | Rowland, M. *et al.* | 2021 | Narrative review | Evaluate crisis management principles and practices in a targeted manner. | Prehospital | Situational awareness | Situational awareness has 3 levels:  - Level 1: Perception of the elements of the environment. This is the first step in achieving SA and concerns the way in which the individual perceives the state, characteristics and dynamics of relevant elements in the environment. In the pre-hospital context, it involves the practitioner's ability to detect and identify diagnostic signs.  - Level 2: Understanding the current situation. Stage 2 goes beyond a simple awareness of the elements present and involves the use of past experience and prior knowledge to integrate and better understand the meaning of these elements in the light of the objectives set. In the pre-hospital context, this requires paramedics to combine signs and symptoms to formulate a possible diagnosis.  - Step 3: Predict the future state. Step 3 builds on steps 1 and 2 and involves the ability to predict and project future problems. This is achieved through knowledge of the state and dynamics of the elements is an understanding of the situation, i.e. after identifying signs and symptoms (Stage 1) that lead to a diagnosis (Stage 2), the practitioner must consider what is likely to happen next (Stage 3). |
| 46 | Hu, X. *et al.* | 2020 | Qualitative study | Define and outline specific non-technical competencies for public health first responders to chemical, biological, radiological and nuclear (CBRN) emergencies in China. | Disaster medicine | Situational awareness, communication skills, collaboration, resource management, task management, cultural competency, austere environment skills, physical stamina. | The following non-technical areas of competence were identified  - Situational awareness  - Communication skills  - Collaboration skills  - Resource management  - Task management  - Cultural Competence  - Strong environmental skills  - Physical stamina  The following sub-domains were identified  o Applied knowledge to detect, predict and identify CBR agents.  o Understanding of the impact of environmental factors on the spread of CBR agents.  o Knowledge of the potential health and environmental effects of typical CBR agents.  o Recognise the limitations of individual and group knowledge, skills and authority.  o Evaluate and plan for the effectiveness of all actions taken.  o Describe the role, channels and processes of communication in response to a CBRN incident.  o Properly use all communication devices while wearing protective equipment in hazardous environments.  o Outline the principles of risk communication in response to CBRN incidents.  o Understand and identify the roles, responsibilities and capabilities of agencies and partners in responding to a CBRN incident.  o Identify and locate agency and partner response plans for CBRN incidents.  o Ability to identify and forecast critical resources required to respond to a CBRN incident.  o Ability to establish processes for mobilising, requesting and managing resources.  o Knowledge and maintenance of policies, guidelines and protocols.  o Adherence to general ethical principles in the emergency context.  o Intercultural respect.  o Flexibility/adaptability. |
| 47 | Lowe, D. J., *et al*. | 2016 | Review | Propose a model to be considered to support the evaluation and training of AS within the ED. | Emergency Department | Situational awareness | A clear and informed understanding of 'what is going on, what it means and what I need to do' is fundamental to the practice of EM. Improving awareness of one's own cognitive process allows one to recalibrate one's approach in the context of the myriad biases and distractions that occur on each shift in the ED.  Three levels of situational awareness have been identified: patient, team and department:  - Quickly and accurately assess the patient. Anticipates rate-limiting steps in care and gathers critical data to facilitate assessment.  - Uses the team effectively and has a global appreciation of skills; acts appropriately. Flexible to perceived challenges.  - Broad awareness of the department. Effective use of skill mix according to need. |
| 50 | Gogalniceanu, P. *et al.* | 2022 | Observational study | To develop a framework for surgical crisis recovery based on problem-solving interventions used by pilots in commercial aviation. | Emergency surgery | Decision making, workload management, situational awareness | 3 strategies: (1) building cognitive capacity by improving situational awareness and workload management; (2) using checklists in abnormal situations to implement emergency operating procedures; (3) making structured decisions using analysis-based problem-solving cycles (T-DODAR framework). |
| 51 | Sterling, M.R. | 2006 | Report | Provide an overview of the SFRM model. | Space flight | Command, leadership, workload management, situation awareness, decision making. | SFRM, developed for spaceflight in collaboration with NASA, has identified 6 areas of non-technical competence:  - Command  - Leadership  - Communication skills  - Workload management  - situational awareness  - Decision-making  Decision making is considered a critical element for the safety of flight and space operations. The decision making process is divided into three phases, represented by a triangle pointing downwards:  - Design/Planning Phase  - Training phase  - Operational phase  The time window for making a decision is reduced in proportion to the phase in which one is working. The phases are called windows of awareness. |
| 52 | Hall, C., *et al*. | 2020 | Trial | Test the effectiveness of TEMPIST (cognitive aid) in a simulated emergency room environment by observing the team's error rates. | Emergency Department | Guidelines, protocols, standards, technical expertise. | This study showed that by following the linear, step-by-step pathways in the handbook, doctors more than halved the error rate of their teams in four simulated medical crises. The manual improves team performance and enables healthcare teams to reduce clinical error rates, thereby reducing harm to patients.  (Seventy-five participants (38 doctors and 37 nurses) were divided into 21 groups. The groups were exposed to 84 simulated crises, resulting in a total of 1260 key tasks. Group size ranged from three to six team members. The majority of groups (n = 13) consisted of three members, followed by five groups of four participants, two groups of five participants and one group of six participants.)  In this simulation-based study, we found that the use of a cognitive aid led to a significant reduction in error rates. All groups in the study reduced their error rates by at least 20% when they had access to the emergency protocol manual. Overall, there was a 54% reduction in errors across all four scenarios. |
| 53 | Flowerdew, L., *et al*. | 2013 | Observational study | Evaluation of a new tool to assess non-technical competencies of emergency physicians. | Emergency Department | Leadership | This study shows that the performance of the assessment tool is acceptable and provides valuable information for structuring the assessment and training of non-technical competences, particularly in relation to leadership. The competency framework can be used to identify areas for development for individual learners. |
| 54 | Ranasinghe, P., *et al*. | 2017 | Cross-sectional study | To explore the relationship between EI, perceived stress and academic performance and related factors in medical students. | Education | Emotional intelligence | Higher EI was associated with better academic performance among final year medical students. Higher EI was also observed in those with higher levels of self-satisfaction. Self-perceived stress was lower in those with higher EI. |
