# Supplementary material for: Emergency team competencies: scoping review for the development of a tool to support the briefing and debriefing activities of emergency healthcare providers
Source: J Anesth Analg Crit Care. 2023 Jul 28;3:24. doi: 10.1186/s44158-023-00109-3 (PMC10386683; doi:10.1186/s44158-023-00109-3)
Supplement: Supplementary file 2 — Additional file 2. Mapping of behavioural markers broken down in the ETC tool. [file 44158_2023_109_MOESM2_ESM.docx]

| **Mapping of behavioural markers broken down in the ETC tool** | | |
| --- | --- | --- |
| Competencies | Area | Elements/Behaviour |
| Decision making | • |  |
| Situation awareness | • |  |
| Task management | • |  |
| Problem solving |  | • |
| Standard |  | • |
| Cognitive flexibility |  | • |
| Communication | • |  |
| Decision aids |  | • |
| Diagnosis |  | • |
| Leadership | • |  |
| Generation of options |  | • |
| Risk assessment |  | • |
| Cooperation | • |  |
| Checking the results |  | • |
| Shared mental models |  | • |
| Teamwork |  | • |
| Analysis |  | • |
| Anticipating |  | • |
| Shared mental models |  |  |
| Stress/fatigue management | • |  |
| Teamwork |  | • |
| Information gathering |  | • |
| Environmental awareness |  | • |
| Scene management |  | • |
| Self-awareness |  | • |
| Team situation awareness |  | • |
| Respect |  | • |
| Data management |  | • |
| Distribution of tasks |  | • |
| Preparing |  | • |
| Prioritizing |  | • |
| Time management |  | • |
| Resource management |  | • |
| Role awareness |  | • |
| Task analysis |  | • |
| Reflection |  | • |
| Workflow assessment |  |  |
| Autority |  | • |
| Assertive |  | • |
| Proactive communication |  | • |
| Clinical skills | • |  |
| Empathy |  | • |
| Professional |  | • |
| Rapport |  | • |
| Ethical |  | • |
| Listening |  | • |
| Clarity and relevance |  | • |
| Circularity |  | • |
| Briefing |  | • |
| Leadership Style |  | • |
| Planning |  | • |
| Transformational leadership |  | • |
| Conflict management |  | • |
| Debriefing |  | • |
| Followership |  | • |
| Sharing |  | • |
| Management |  | • |
| Teamwork |  | • |
| Supporting others |  | • |
| Sharing |  | • |
| Request for help |  | • |
| Manages pressure |  | • |
| Emotional Intelligence |  | • |
| Resilience |  | • |
| Coping strategies |  | • |
| Stress factors |  | • |
| System awareness |  | • |
| Knowledge and application of procedures/guidelines |  | • |
| Understanding of legal regulations |  | • |
| Quick look |  | • |
