# Supplementary material for: Emergency team competencies: scoping review for the development of a tool to support the briefing and debriefing activities of emergency healthcare providers
Source: J Anesth Analg Crit Care. 2023 Jul 28;3:24. doi: 10.1186/s44158-023-00109-3 (PMC10386683; doi:10.1186/s44158-023-00109-3)
Supplement: Supplementary file 3 — Additional file 3. Emergency Team Competencies tool. [file 44158_2023_109_MOESM3_ESM.docx]

| **EMERGENCY TEAM COMPETENCIES TOOL** | |
| --- | --- |
| **AREAS** | **ELEMENTS** |
| **COMMUNICATION** | **Listening**  **Clarity and relevance**  **“Close loop”**  **Circular feedback**  **Standard phraseology**  **Assertiveness** |
| **DECISION MAKING** | **Diagnosis**  **Options**  **Risk Assessment**  **Review** |
| **CLINICAL SKILLS** | **System awareness**  **Compliance with standards**  **Knowledge and application of procedures/guidelines**  **Understanding of legal regulations**  **Quick look** |
| **SITUATION AWARENESS** | **Information gathering**  **Understanding and analysis**  **Anticipation** |
| **LEADERSHIP** | **Leadership style**  **Briefing and Planning**  **Conflict management** |
| **TASK MANAGEMENT** | **Priorities setting**  **Time optimization**  **Resource management**  **Tasks distribution**  **Role adherence** |
| **COOPERATION** | **Coordination and Integration**  **Supporting others**  **Sharing**  **Call for help** |
| **STRESS & FATIGUE MANAGEMENT** | **Stress factors**  **Emotions**  **Coping strategies** |
