# Supplementary material for: Emergency team competencies: scoping review for the development of a tool to support the briefing and debriefing activities of emergency healthcare providers
Source: J Anesth Analg Crit Care. 2023 Jul 28;3:24. doi: 10.1186/s44158-023-00109-3 (PMC10386683; doi:10.1186/s44158-023-00109-3)
Supplement: Supplementary file 4 — Additional file 4. Mapping of the behavioural markers, elements and competence domains explored in the included studies and multistage development of the ETC tool. [file 44158_2023_109_MOESM4_ESM.docx]

Mapping of the behavioural markers, elements, competence domains explored in the included studies and multi-stage development of the ETC tool

| **Mapping of the behavioural markers, elements, competence domains explored in the included studies and multi-stage development of the ETC tool** | | | | | | | | | | |
| --- | --- | --- | --- | --- | --- | --- | --- | --- | --- | --- |
| Competencies explored | Phase 1: literature | Elements explored | First Round | | Second Round | | Third Round | | Fourth Round | |
|  |  |  | Inclusion | Exclusion | Inclusion | Exclusion | Inclusion | Exclusion | Inclusion | Exclusion |
| Decision making | [16][30][36][37][38][44][46][48]  [49][50][51] | Problem solving | • |  | ∆ | | ∆ | | ∆+ | |
|  |  | Standard | ο | | ο | | ο | | • |  |
|  |  | Cognitive flexibility | • |  | ∆ | | ∆ | | ∆+ | |
|  |  | Decision aids | • |  | ∆ | | ∆ | | ∆+ | |
|  |  | Diagnosis | • |  | ∆ | | ∆ | | ∆+ | |
|  |  | Generation of options | • |  | ∆ | | ∆ | | ∆+ | |
|  |  | Risk assessment | • |  | ∆ | | ∆ | | ∆+ | |
|  |  | Checking the results | • |  | ∆ | | ∆ | | ∆+ | |
|  |  | Shared mental models | ο | | ο | | • |  | ∆+ | |
|  |  | Teamwork | • |  | ∆ | | ∆ | | ∆+ | |
| Situation awareness | [16][22][29][30][36][37][38][39][41]  [42][43][44][45][47] | Information gathering | • |  | ∆ | | ∆ | | ∆+ | |
|  |  | Analysis | • |  | ∆ | | ∆ | | ∆+ | |
|  |  | Anticipating | • |  | ∆ | | ∆ | | ∆+ | |
|  |  | Shared mental models | ο | | ο | | • |  | ∆+ | |
|  |  | Environmental awareness | ο | | ο | | ο | | • |  |
|  |  | Scene management | ο | | ο | | ο | | • |  |
|  |  | Self-awareness | ο | | ο | | • |  | ∆+ | |
|  |  | Team situation awareness | ο | | ο | | ο | | • |  |
|  |  | Time management | ο | | ο | | ο | | • |  |
|  |  | Data management | • |  | ∆ | | ∆ | | ∆+ | |
| Task management | [12][27][34][35][36][37][38][39] | Distribution of tasks | • |  | ∆ | | ∆ | | ∆+ | |
|  |  | Preparing | • |  | ∆ | | ∆ | | ∆+ | |
|  |  | Prioritizing | ο | |  | | • |  | ∆+ | |
|  |  | Time management | ο | | • |  | ∆ | | ∆+ | |
|  |  | Resource management | • |  | ∆ | | ∆ | | ∆+ | |
|  |  | Role awareness | ο | | • |  | ∆ | | ∆+ | |
|  |  | Task analysis | • |  | ∆ | | ∆ | | ∆+ | |
|  |  | Reflection | ο | | ο | |  | • | — | |
|  |  | Workflow assessment | • |  | ∆ | | ∆ | | ∆+ | |
| Communication | [9][10][11][12][13][14][15][16][17]  [18][19][20] | Autority | • |  | ∆ | | ∆ | | ∆+ | |
|  |  | Assertive | • |  | ∆ | | ∆ | | ∆+ | |
|  |  | Proactive communication | ο | | ο | | • |  | ∆+ | |
|  |  | Respect | ο | | • |  | ∆ | | ∆+ | |
|  |  | Empathy | ο | | • |  | ∆ | | ∆+ | |
|  |  | Professional | • |  | ∆ | | ∆ | | ∆+ | |
|  |  | Rapport | ο | |  | • | — | | — | |
|  |  | Ethical | • |  | ∆ | | ∆ | | ∆+ | |
|  |  | Listening | ο | | ο | | ο | | • |  |
|  |  | Clarity and relevance | • |  | ∆ | | ∆ | | ∆+ | |
|  |  | Circularity | • |  | ∆ | | ∆ | | ∆+ | |
|  |  | Standard | • |  | ∆ | | ∆ | | ∆+ | |
| Leadership | [9][10][15][19][20][21][22][23][24]  [25][26][27][28][29][30][31][32]  [33][34] | Briefing | • |  | ∆ | | ∆ | | ∆+ | |
|  |  | Leadership Style | ο | | ο | | • |  | ∆+ | |
|  |  | Planning | • |  | ∆ | | ∆ | | ∆+ | |
|  |  | Transformational leadership | ο | | ο | |  | • | — | |
|  |  | Conflict management | • |  | ∆ | | ∆ | | ∆+ | |
|  |  | Debriefing | ο | | ο | | ο | | • |  |
|  |  | Followership | ο | | ο | | ο | |  | • |
|  |  | Respect | ο | | ο | | ο | | • |  |
|  |  | Sharing | • |  | ∆ | | ∆ | | ∆+ | |
| Cooperation | [11][22][30][40][41] | Management | • |  | ∆ | | ∆ | | ∆+ | |
|  |  | Teamwork | • |  | ∆ | | ∆ | | ∆+ | |
|  |  | Supporting others | • |  | ∆ | | ∆ | | ∆+ | |
|  |  | Sharing | ο | | • |  | ∆ | | ∆+ | |
|  |  | Request for help | • |  | ∆ | | ∆ | | ∆+ | |
| Stress/fatigue management | [43][44][53][54] | Manages pressure | • |  | ∆ | | ∆ | | ∆+ | |
|  |  | Empathy | ο | | ο | | • |  | ∆+ | |
|  |  | Emotional Intelligence | ο | | ο | |  | | • |  |
|  |  | Resilience | • |  | ∆ | | ∆ | | ∆+ | |
|  |  | Coping strategies | ο | | • |  | ∆ | | ∆+ | |
|  |  | Stress factors | • |  | ∆ | | ∆ | | ∆+ | |
| Clinical skills | [9][30] | System awareness | ο | | ο | | • |  | ∆+ | |
|  |  | Standard | • |  | ∆ | | ∆ | | ∆+ | |
|  |  | Knowledge and application of procedures/guidelines | • |  | ∆ | | ∆ | | ∆+ | |
|  |  | Understanding of legal regulations | ο | | ο | | • |  | ∆+ | |
|  |  | Quick look | ο | | ο | | ο | | • |  |

Legend : • Inclusion/Exclusion ο Not yet investigated ∆ Under review ∆+ Review confirmed ∆— Revision not confirmed — Item excluded
